# A Flame-Retardant Cyclophosphazene as an Electrolyte Component for Si-Graphite Anodes for Lithium-Ion Batteries

**DOI:** 10.3390/ijms27010028

**Published:** 2025-12-19

**Authors:** Yulia Vlasova, Sergei Potapov, Mikhail Kokontsev, Shakhboz Isokjanov, Olesia Karakulina, Alena Komayko, Alina Inozemtseva, Viacheslav Savin, Lidiya Minaeva, Alexandra Ageshina, Aleksandra Rzhevskaia, Valery Krivetskiy

**Affiliations:** 1 Moscow Center for Advanced Studies, Moscow 123592, Russia; iuliia.vlasova@chemistry.msu.ru (Y.V.); qgenwat@yandex.ru (S.P.); cvoyya@gmail.com (M.K.); isakjanov2997@gmail.com (S.I.); komaykoa@gmail.com (A.K.); a.inozemtseva@chph.ras.ru (A.I.); minaeva.lidiya@gmail.com (L.M.); ageshinaaa@yandex.ru (A.A.); rzhevskaia.a@gmail.com (A.R.); 2 Chemistry Department, Lomonosov Moscow State University, Moscow 119991, Russia; 3 Physics Department, Lomonosov Moscow State University, Moscow 119991, Russia; 4 N.N. Semenov Federal Research Center for Chemical Physics, Moscow 119991, Russia; v.savin@chph.ras.ru

**Keywords:** cyclophosphazene, electrolyte additive, flame retardant, Si/C composite, lithium-ion batteries, solid–electrolyte interphase (SEI), cycling stability

## Abstract

Silicon-graphite anodes offer a practical route to increase the energy density of lithium-ion batteries (LIBs), but their widespread adoption is hampered by cyclic instability due to huge volume changes of silicon during lithiation/delithiation process. Another fallout of LIBs capacity gain is growing safety concerns due to fire risks, associated with the uncontrolled release of chemical energy. Herein, we test a hexakis(fluoroethoxy)phosphazene (HFEPN) as a multifunctional electrolyte additive designed to mitigate both issues. The flammability of HFEPN-containing electrolytes was evaluated using a self-extinguishing time test, while the electrochemical performance was assessed in Si/C composite||NMC pouch cells under a progressively accelerated cycling protocol. It is shown that the additive fully imparts flame-retardant properties to the electrolyte at a 15 wt% loading. Despite forming a more stable solid–electrolyte interphase (SEI) with enhanced interfacial kinetics the additive did not improve the cycling stability of the Si/C-based cells. The cells with 15 wt% HFEPN retained 43% of capacity after 70 cycles, comparable to 46.5% for the reference electrolyte. The diffusion limitations imposed by the increased electrolyte viscosity are assumed to offset the interfacial benefits of the additive. Thus, alongside the improved synthetic route, this study demonstrates that while HFEPN functions as an effective flame retardant and SEI modifier, its practical benefits for silicon anodes are limited at high concentrations by detrimental effects on electrolyte transport properties and should be improved in future molecular design efforts.

## 1. Introduction

Li-ion batteries (LIBs) are in high demand for energy storage applications, portable devices, and electric vehicles, driving a constant need for improving their energy density. Since lithium cobalt oxide (LCO) was introduced, energy density has been primarily increased through the development of new cathode materials. Today, the potential for further improvement via the cathode is diminishing. Therefore, a further increase in the specific energy of LIBs is now to a great extent associated with the technology of the negative electrode [1,2,3]. The two main directions of development are the use of lithium-metal anodes or so-called anode-free batteries [1,4] on the one hand, and the use of high-capacity silicon-containing anodes on the other [5,6,7,8]. The latter technology development direction offers a path for smooth practical implementation [9,10,11] because the same production facilities as for conventional graphite-anode LIBs can be used [12,13]. Consequently, numerous Si-graphite composite anode materials have become commercially available lately [14].

The widespread application of Si-containing anode-based LIBs is hampered by a crucial obstacle, namely, the inherent instability of silicon particles, caused by their huge (~300%) volume expansion [15,16] and contraction upon lithiation and delithiation [17]. The use of nanosized Si particles alongside with specialized binders, carbon nanotube and graphene additives [6,11,12], as well as three-dimensional current collectors is considered as a powerful means to mitigate the negative effects of silicon particle size alteration [18] and to tame the anode capacity degradation [19]. Another significant factor of Si-anode stabilization is the formation of a thin, but strong and elastic solid–electrolyte interphase (SEI) on the surface of anode particles, which can be achieved with the help of polymerizing components of electrolyte [20,21].

At the same time, the increase in the energy density of modern LIBs raises serious safety concerns during their practical applications [22,23,24]. The uncontrolled release of this stored energy due to cell damage or thermal runaway can lead to intense fires.

Thus, the incorporation of flame-retardant components into LIBs with high energy density is becoming increasingly important [25,26,27,28].

Cyclophosphazenes, such as hexakis(fluoroethoxy)phosphazene (HFEPN), are well-known for their flame-retarding properties, which are attributed to their high phosphorus and nitrogen content [29]. Intriguingly, recent studies have also suggested their potential to participate in the formation of a stable solid–electrolyte interphase on high-capacity anodes [30,31,32]. This proposed dual functionality—simultaneously mitigating flammability and stabilizing the silicon interface—is highly appealing.

In this work, we test the dual functionality of a cyclophosphazene molecule as both a flame retardant and an SEI-stabilizing additive for Si-containing anodes.

## 2. Results

### 2.1. Synthesis of HFEPN

The approaches for preparing hexakis(2,2,2-trifluoroethoxy)cyclotriphosphazene are based on the exhaustive nucleophilic substitution reaction of hexachlorocyclotriphosphazene with trifluoroethoxide. A common method involves the reaction of hexachlorocyclotriphosphazene and sodium trifluoroethanol in diethyl ether [33,34]. The authors report that when attempting to isolate hexakis(2,2,2-trifluoroethoxy)cyclotriphosphazene by distillation or sublimation in a vacuum, they frequently observed explosions. It was hypothesized that the explosive decomposition of the product at high temperatures could be caused by traces of alkaline compounds, in particular by the residual sodium trifluoroethoxide.

To ensure a safer and higher-yielding process, we developed an alternative synthetic route to obtain hexakis(2,2,2-trifluoroethoxy)cyclotriphosphazene. Tetrahydrofuran (THF) was chosen as the solvent for its superior dissolving power in comparison to diethyl ether and a higher boiling point. First, sodium trifluoroethoxide was prepared by dropwise adding trifluoroethanol to sodium metal in anhydrous THF. After complete dissolution of the sodium, a solution of hexachlorocyclotriphosphazene in THF was slowly added to the reaction mixture. The use of a slight excess of trifluoroethanol ensured complete substitution and facilitated the subsequent purification. The crude product was extracted into dichloromethane, and the organic solution was washed with water to remove alkaline impurities. Final purification by recrystallization from a pentane/diethyl ether mixture afforded pure hexakis(2,2,2-trifluoroethoxy)cyclotriphosphazene as a white solid in 87% yield.

**Figure A1:**
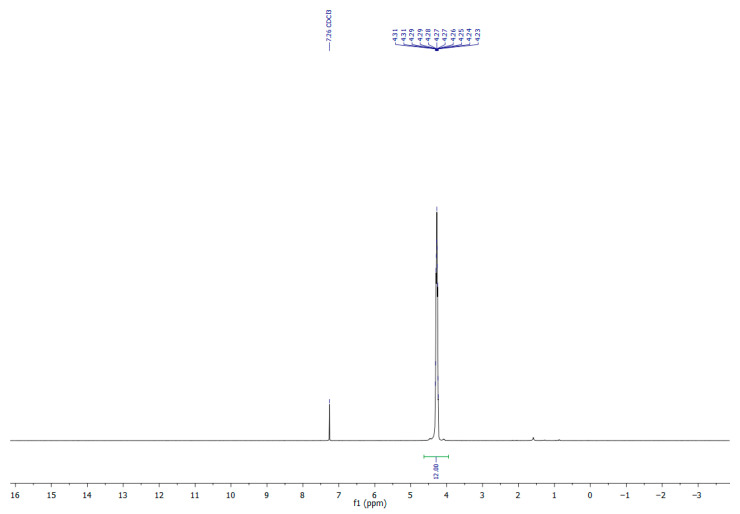
^1^H NMR (400 MHz, Chloroform-d) of Hexakis(2,2,2-trifluoroethoxy)cyclotriphosphazene.

**Figure A2:**
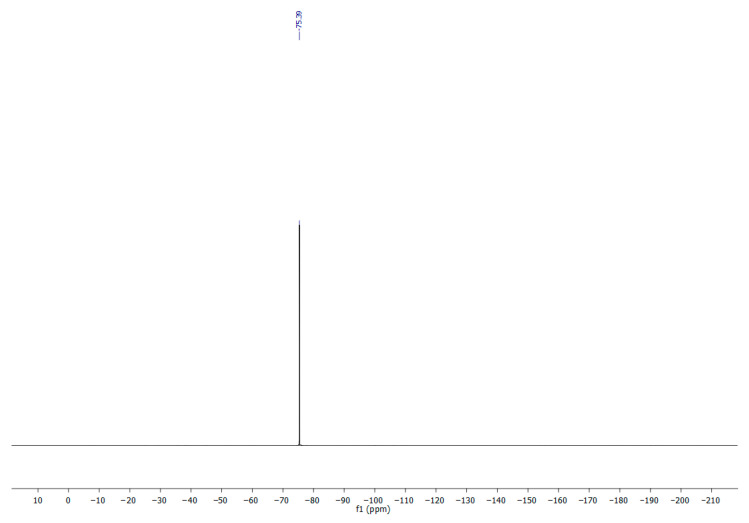
^19^F{1H} NMR (376 MHz, Chloroform-d) of Hexakis(2,2,2-trifluoroethoxy)cyclotriphosphazene.

**Figure A3:**
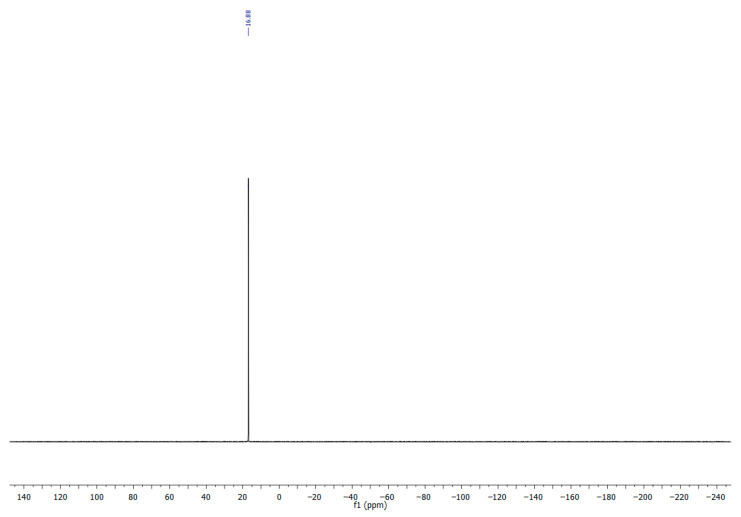
^31^P{1H} NMR (162 MHz, Chloroform-d) of Hexakis(2,2,2-trifluoroethoxy)cyclotriphosphazene.

**Figure A4:**
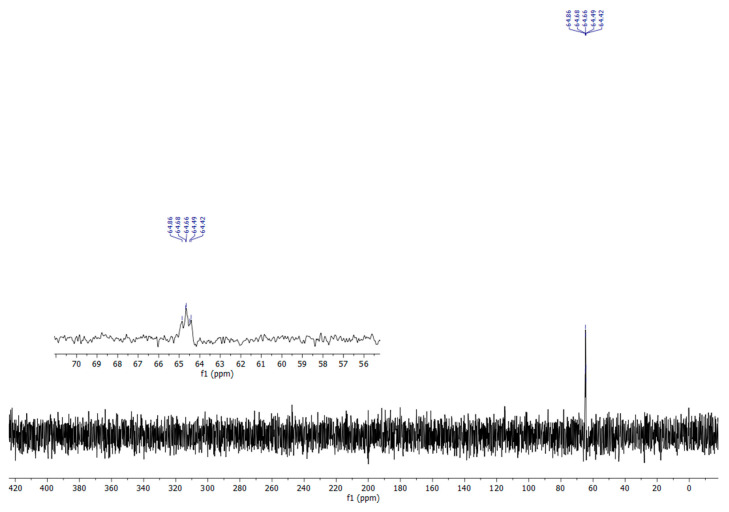
^15^N NMR (40 MHz, Chloroform-d) of Hexakis(2,2,2-trifluoroethoxy)cyclotriphosphazene.

**Figure A5:**
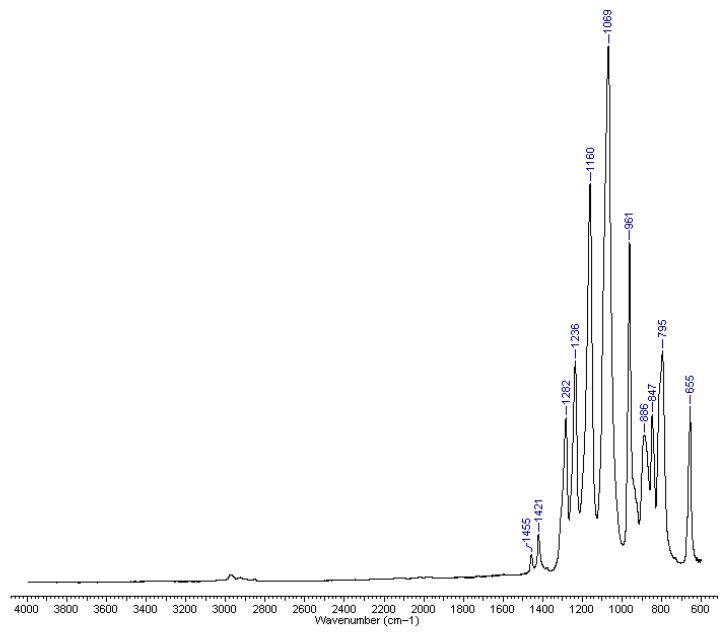
IR spectra of Hexakis(2,2,2-trifluoroethoxy)cyclotriphosphazene.

The successfully synthesized product was further characterized using NMR and IR spectroscopy. The ^19^F and ^31^P NMR spectra showed characteristic singlet peaks at −75.39 ppm and 16.9 ppm, respectively. In the ^1^H NMR spectrum, a multiplet peak at 4.2–4.4 ppm corresponded to the methylene protons of the trifluoroethoxy groups. A signal was also observed in the ^15^N NMR spectrum at 64.7 ppm (see Appendix A, Figure A1, Figure A2, Figure A3, Figure A4 and Figure A5).

### 2.2. Physicochemical Properties and Electrochemical Stability of Electrolytes

The purified and dried HFEPN was transferred to an Ar-filled glovebox. A reference electrolyte consisting of 1 M LiPF_6_ in ethylene carbonate/ethyl methyl carbonate (EC/EMC, 1:3 *v*/*v*) with 1 wt% vinylene carbonate (VC) was employed. A series of electrolyte blends were prepared by incorporating HFEPN at concentrations of 5, 10, and 15 wt%, while maintaining a constant LiPF_6_ concentration of 1 M.

**Figure 1:**
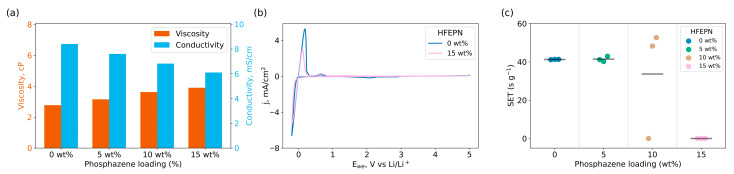
(**a**) Viscosity and ionic conductivity of electrolytes with different HFEPN concentrations. (**b**) Cyclic voltammograms of the reference electrolyte and the electrolyte with 15 wt% HFEPN (Pt disk electrode, sweep rate 5 mV/s). (**c**) Self-extinguishing time (SET) of electrolytes with different HFEPN concentrations.

The effects of HFEPN concentration on the physical properties of the electrolyte are summarized in Figure 1a. The viscosity of the reference electrolyte is 2.7 cP. A systematic increase in viscosity was observed with increasing HFEPN content, reaching approximately 3.9 cP at the maximum loading of 15 wt%. Concomitantly, the ionic conductivity decreased from 8.4 mS/cm for the reference electrolyte to approximately 6.1 mS/cm for the 15 wt% HFEPN mixture. This reduction in conductivity is primarily attributed to the increased viscosity imparted by the phosphazene additive, which hinders the mobility of Li^+^ ions in the electrolyte, according to the Stokes-Einstein relation [35,36]. Numerical values of viscosity and ionic conductivity measurements are provided in Table A1.

Figure 1b shows the CV curves for the reference electrolyte and the same electrolyte with 15 wt% HFEPN, measured between −0.2 and 5.0 V (vs. Li/Li^+^). The results indicate that the additive does not alter the electrolyte’s oxidative or reductive stability. The voltammograms show only Li plating/stripping faradaic currents near 0 V vs. Li/Li^+^. Furthermore, no new faradaic peaks appear in the 0.5–5.0 V range, confirming the electrochemical stability of the phosphazene additive.

### 2.3. Flame Retarding Properties

The carbonate solvents enable high ionic conductivity of LIBs, but present a significant safety concern being inherently volatile and flammable. To mitigate this flammability, HFEPN was evaluated as a flame-retardant additive. Its efficacy was quantified by measuring the self-extinguishing time (SET); the results are presented in Figure 1c. An electrolyte sample (approx. 0.8 g) with or without HFEPN (5, 10, or 15 wt%) was soaked into a 4 cm piece of silica fiber wick. The flame source was removed upon ignition. Contrary to the typical monotonic decrease in SET with increasing additive concentration [37,38], a distinct threshold behavior was observed. Combustion persisted consistently up to 10 wt% HFEPN. At 10 wt%, the combustion became erratic, with some tests resulting in extinguishment and others in sustained burning (as visually documented in Supplementary Video S1), yet the average SET remained largely unchanged. A complete suppression of combustion was achieved only at 15 wt% HFEPN. Consequently, this concentration was selected as the upper limit for further electrochemical testing.

### 2.4. Electrochemical Performance in Full Cells

The impact of HFEPN at different concentration (0, 5, 10, and 15 wt%) on electrochemical performance was studied in full cells of two types: NMC811||graphite (Gr cells) and NMC811||Si/C composite (Si/C cells).

The electrochemical performance was evaluated following an initial formation protocol. All cycling was performed within a voltage window of 2.7 V to 4.2 V. Graphite-based cells underwent a rate capability test (20 cycles each at 0.1C, 0.2C, and 0.4C), while silicon-based cells were subjected to a more strenuous protocol to probe degradation (10 cycles at 0.1C, 20 cycles at 0.2C, 40 cycles at 0.4C, and a final 10 cycles at 0.1C to assess capacity recovery). All data reported herein corresponds to the post-formation cycling phase and represents the average performance of three replicate cells.

**Figure 2:**
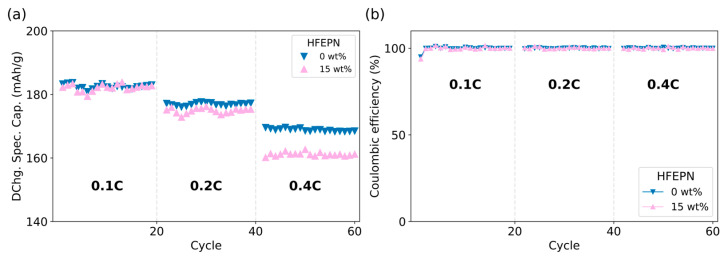
Cycling performance of NMC811||Graphite pouch cells with different HFEPN concentrations: (**a**) Discharge specific capacity showing a rate-dependent decrease. (**b**) Coulombic efficiency.

The electrochemical performance of graphite-based cells is summarized in Figure 2. At a low current rate of 0.1C, the discharge specific capacity was nearly identical for cells with and without the phosphazene additive, with an average value of 182 mAh/g (Figure 2a). However, a performance divergence emerged at higher current rates. At 0.2C cells containing 15 wt% HFEPN exhibited a slightly lower capacity (175 mAh/g) compared to the reference electrolyte (177 mAh/g). This difference became more pronounced at 0.4C (161 mAh/g vs. 169 mAh/g for the reference electrolyte). This rate-dependent performance loss is consistent with the higher viscosity and lower ionic conductivity of the HFEPN-containing formulations. Despite this, no degradation of the graphite anode material itself was observed, and the Coulombic efficiency for all cells approached 100% (Figure 2b).

**Figure 3:**
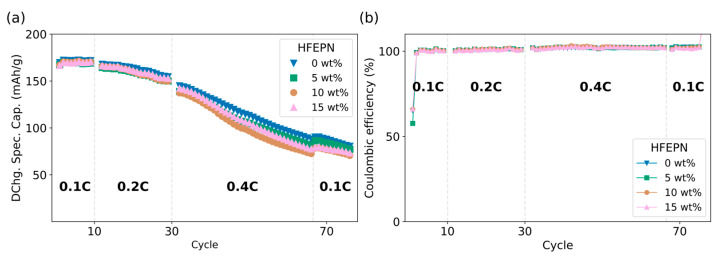
Electrochemical performance of NMC811||Si/C composite full cells: (**a**) Discharge specific capacity illustrating a progressive fade during high-rate (0.4C) cycling and limited recovery upon returning to 0.1C. (**b**) Coulombic efficiency.

The cycling performance of the Si/C-based cells is presented in Figure 3. At the initial 0.1C rate, all cells performed similarly, with an average discharge capacity of 170 mAh/g and no significant divergence based on HFEPN loading. A marked capacity fade was observed upon switching to 0.2C, with capacities dropping to the 154–150 mAh/g range. This degradation was exacerbated during extended cycling at 0.4C, where the discharge capacity was reduced to approximately half of its initial value (88–68 mAh/g, Figure 3a).

When the current rate was returned to 0.1C, the cells could only recover to roughly half of their starting capacity (80–70 mAh/g), indicating profound and irreversible degradation. The performance differences between the electrolytes, while clear during the high-rate stress, became less pronounced at this final stage. The coulombic efficiency for all silicon-based cells approached 100%, with minor fluctuations likely attributable to uncontrolled minor variations in ambient conditions (Figure 3b).

**Figure 4:**
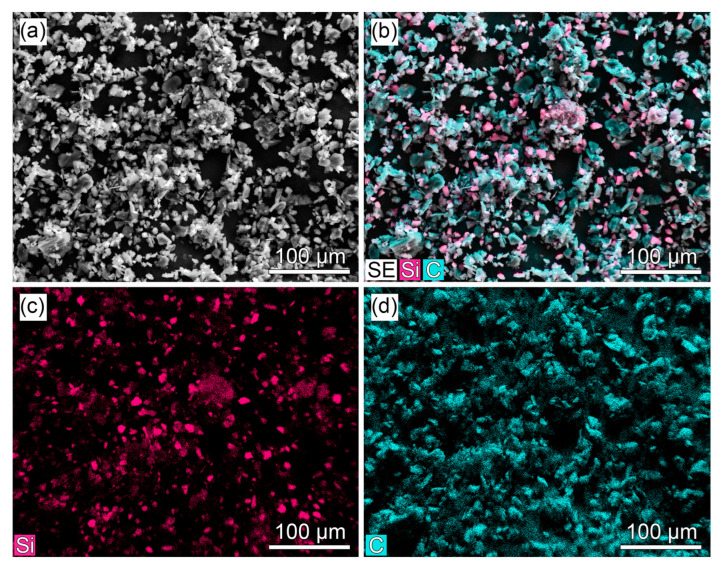
Morphology and elemental composition of the DXB8 Si/C composite anode material. (**a**) Representative SEM image. (**b**) Combined EDX elemental map. Separate EDX maps for (**c**) silicon Si and (**d**) carbon C. The maps show the dispersion of silicon particles within a carbonaceous matrix.

The morphology and elemental distribution of the commercial DXB8 Si/C composite anode powder were characterized using scanning electron microscopy (SEM) with energy-dispersive X-ray spectroscopy (EDX). The results are presented in Figure 4. The SEM image (Figure 4a) shows a particulate morphology typical of such composites, with a broad particle size distribution. The combined EDX map (Figure 4b) and the separate elemental maps for silicon (Si, Figure 4c) and carbon (C, Figure 4d) confirm the uniform dispersion of silicon domains within a continuous carbon-based matrix. The silicon particles appear as fine, discrete crystallites embedded in the carbon structure.

### 2.5. Electrochemical Impedance Spectroscopy (EIS)

**Figure 5:**
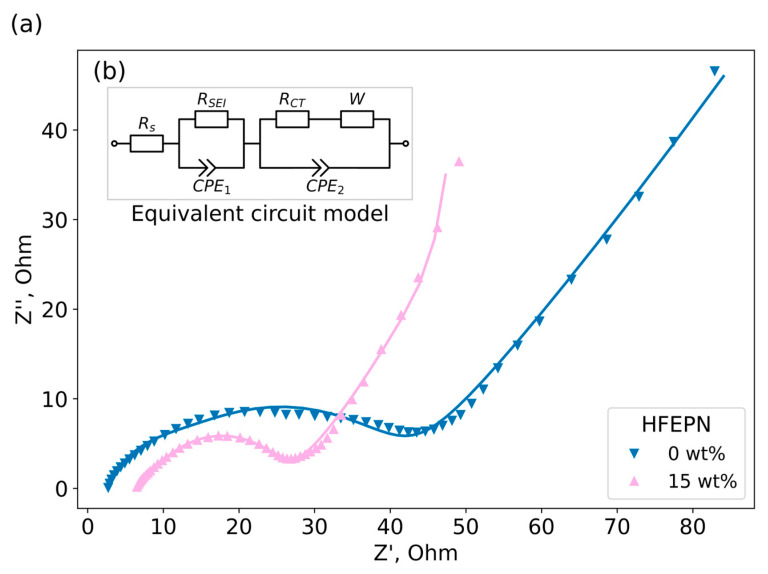
(**a**) Nyquist plots from electrochemical impedance spectroscopy of Si/C composite||Li coin cells at 0.7 V vs. Li/Li^+^. Inset (**b**): Equivalent circuit used to fit the experimental EIS plots, where R_S_—resistance of the solution, CPE_1_ and CPE_2_—constant phase elements, R_SEI_—resistance of solid electrolyte interphase, R_CT_—charge transfer resistance, W—Warburg element.

To characterize the impact of HFEPN on the kinetics of the interfacial charge transfer process, electrochemical impedance spectroscopy was performed on Si/C composite||Li half-cells after one formation cycle. The Nyquist plots (Figure 5a) were fitted using an equivalent circuit model (Figure 5b) to quantify the interfacial resistances. The analysis revealed that the addition of 15 wt% HFEPN led to a drastic reduction in charge transfer resistance (R_CT_), thus facilitating interfacial kinetics compared to the reference electrolyte. The ability of phosphazene additives to improve interphase behavior and provide flame retardancy at the same time indicates them as promising multifunctional electrolyte components.

## 3. Discussion

An increase in viscosity and a decrease in ionic conductivity align well with established literature. As anticipated for the introduction of a solid compound into a liquid electrolyte, a marked concentration-dependent increase in viscosity was observed, which is a typical behavior for high molar mass molecules such as HFEPN (~730 g/mol) [39,40]. This increase in viscosity is directly correlated with a concomitant decrease in ionic conductivity. The underlying mechanism is attributed to the slight coordination of Li^+^ ions by HFEPN [41], which reduces lithium-ion mobility and thus diffusivity [42]. Consequently, a concentration-dependent relationship is evident, whereby higher additive loadings lead to greater viscosity and lower ionic conductivity [43]. This trade-off between safety function and transport properties is a well-documented characteristic of flame-retardant additives in Li-ion batteries [44]. Notably, a 15 wt% loading–is sufficient to achieve the desired flame-retardant efficacy—the relative increase in viscosity and the decrease in ionic conductivity remain manageable at approximately 40%, which is consistent with previous studies [38,41].

Furthermore, HFEPN demonstrated a wide electrochemical stability window up to 5 V vs. Li/Li^+^ (Figure 1b), which is a key prerequisite for its use in high-voltage lithium-ion battery applications. These findings are in strong agreement with linear sweep voltammetry (LSV) data reported elsewhere [29,39,40]. The additive’s thermal stability has also been established in the literature through prolonged storage in a thermal chamber [39], as well as differential scanning calorimetry (DSC) and cycling experiments at elevated temperatures [29]. The combination of these properties—wide electrochemical stability and robust thermal performance—validates the application of HFEPN as a safety-enhancing additive for high-voltage, high-temperature lithium-ion batteries.

Regarding the primary function of HFEPN as a flame retardant, our findings reveal a more complex behavior than may be suggested on the base of the published research. Conventional models indicate that the self-extinguishing time (SET) should decrease monotonically with increasing additive concentration [45,46]. However, our results deviate from this expected trend. The standard flammability classification—which categorizes systems as flammable (>20 s/g), flame-retarded (6–20 s/g), or non-flammable (<6 s/g) [31,47]—proved adequate for our system. Experimentally, we observed a sharp transition: the electrolyte remained flammable at 10 wt% HFEPN but became non-flammable at 15 wt% and above.

This deviation from the expected monotonic trend can be explained by our experimental conditions, specifically the use of a larger electrolyte volume compared to standard screening tests [29,46]. This approach likely provides a more realistic simulation of combustion in an actual battery. Under these conditions, the SET did not decrease gradually but exhibited a sharp, step-like change at a specific concentration threshold. This transition point led to challenges in achieving reproducible results within a critical concentration range. This threshold behavior at the flammability limit, rather than a progressive improvement, is consistent with observations for other vapor-phase flame retardants in battery electrolytes [31,48,49] and stems from the vapor-phase action of the flame retardant. The transition is sharp because combustion is sustained by solvent vapors, and the less volatile HFEPN only reaches a critical concentration in the vapor phase after a certain bulk loading is exceeded. The complex, non-equilibrium evaporation from the multicomponent mixture makes the system’s response highly sensitive and difficult to reproduce precisely within a narrow concentration range. This inherent variability in the critical transition region explains the erratic combustion and the less reproducible, sometimes unexpectedly high, SET values observed at 10 wt% HFEPN, as visually documented in Supplementary Video S1. Consequently, test results within this narrow window should be interpreted with caution.

We conclusively determined that consistent ignition suppression was achieved solely at 15 wt% HFEPN. This required concentration is notably higher than the 5–10 wt% typically sufficient in other phosphazene studies [29,38,46]. We attribute this discrepancy primarily to the distinct composition and heightened flammability of our reference electrolyte. A comparative analysis reveals a critical difference: the previously reported formulations, which achieved flame retardancy at lower additive loadings, contained a significantly higher proportion of high-flash-point solvents, notably ethylene carbonate (EC, flash point 143 °C), constituting at least one-third of the solvent volume [29,38]. In contrast, our EC:EMC (1:3 *v*/*v*) formulation contains only 25 vol% EC and is enriched with the highly flammable ethyl methyl carbonate (EMC, flash point 24 °C) [50]. Given the low flash points of the linear carbonates—EMC (24 °C), DMC (16 °C), and DEC (25 °C)—our electrolyte possesses an inherently elevated baseline flammability. This more challenging combustion environment likely necessitates a higher loading of the phosphazene additive to exceed the critical threshold for effective vapor-phase interference and achieve the desired flame-retardant effect.

The electrochemical performance of the NMC811||Gr cells highlights the mass-transport limitations imposed by the HFEPN additive. The near-identical discharge capacity at 0.1C across all formulations indicates that the additive does not detrimentally affect the well-understood intercalation mechanism of graphite or the initial SEI formation. This is consistent with graphite’s minimal volume expansion and its ability to form a stable SEI even with the reference electrolyte. The divergence in performance at higher C-rates (0.2C and 0.4C) is a direct consequence of the altered electrolyte transport properties discussed above. The absence of anode degradation and the consistently high Coulombic efficiency (~100%) confirm that HFEPN does not introduce parasitic reactions at the graphite interface. The primary failure mode in these cells is therefore purely mass-transport polarization, not interfacial instability.

The NMC811||Si/C composite cells, despite their heightened sensitivity to electrolyte composition, revealed a complex response to the HFEPN additive. The similar initial performance at 0.1C suggests that Li^+^ supply is sufficient under mild conditions. However, the applied protocol, particularly the extended cycling at 0.4C, acted as a stress test that revealed profound degradation.

The significant capacity fade and the poor recovery upon returning to 0.1C are characteristic of mechanical and interfacial failure in silicon. We propose a mechanism driven by electrolyte viscosity: the higher viscosity of HFEPN-containing electrolytes hinders homogeneous Li^+^ diffusion during high-rate cycling, creating localized current hotspots on the Si/C anode surface [47,48]. Coupled with silicon’s large volume changes, this inhomogeneous lithiation causes particle cracking and SEI layer pulverization.

This continuous SEI reformation consumes cyclable lithium, resulting in “dead Li” formation, a well-documented failure mode for Si-anodes [49,50]. While phosphazenes can promote a more robust SEI on Si/C composite anodes [31,51], our results suggest that at high concentrations (10–15 wt%), the mass-transport penalty outweighs the potential interfacial benefits. The additive’s ability to form a stable SEI is outpaced by the damage from combined mechanical and kinetic stress.

The overall cell kinetics under high-rate stress are dictated by a critical competition between the opposing factors of improved interfacial charge transfer and hindered bulk ion transport introduced by 15% HFEPN. Although the additive reduces interfacial resistance, forming a more conductive SEI, this benefit is conditional on sufficient Li^+^ supply. At high C-rates, the dominant mass-transport limitation creates a severe Li^+^ flux bottleneck to the Si/C anode surface. This leads to localized current hotspots and inhomogeneous lithiation, which mechanically disrupt the initially improved but fragile interphase during silicon’s volume changes. Thus, the continuous fracture and reformation of the SEI consume cyclable lithium, rendering the interfacial advantage transient. Ultimately, under these strenuous conditions, the detrimental bulk transport properties trump the beneficial interfacial chemistry.

This kinetic analysis not only reconciles the data but also provides robust electrochemical evidence for the poor mechanical resilience of the HFEPN-derived SEI under high-rate stress. The EIS data offer a static snapshot of a fresh, low-resistance interphase formed under mild conditions. Conversely, the high-rate cycling protocol serves as a dynamic stress test. The profound capacity fade and the poor capacity recovery upon returning to a low rate are direct functional indicators of progressive, irreversible lithium loss. Crucially, the sustained high Coulombic efficiency (~100%) rules out continuous electrolyte decomposition as the primary failure mode. Instead, it supports the mechanism of lithium being irreversibly sequestered as “dead Li” due to the continuous fracture and reformation of the SEI layer and the electrical isolation of silicon particles. Therefore, while HFEPN successfully modifies the initial interfacial chemistry, the resulting interphase lacks the robustness to withstand the coupled electrochemical and mechanical strains of high-rate operation on a volume-changing silicon composite anode.

## 4. Materials and Methods

### 4.1. Materials

Hexakis(fluoroethoxy)phosphazene (HFEPN) was synthesized in-house. Commercial precursor, 2,2,4,4,6,6-hexachlorocyclotriphosphazene (Rushim, Moscow, Russia), and sodium (Sigma-Aldrich, St. Louis, MO, USA) and 2,2,2-trifluoroethanol (P&M Invest, Moscow, Russia) were used without further purification.

The reference electrolyte, 1 M LiPF_6_ in ethylene carbonate (EC)/ethyl methyl carbonate (EMC) (1:3 *v*/*v*) with 1 wt% vinylene carbonate (VC), was purchased from Guangzhou Tinci Materials Technology Co. (Guangzhou, China). LiPF_6_ salt (for concentration adjustment) was obtained from Shanshan Technology (Shanghai, China). The cathode active material, LiNi_0.8_Mn_0.1_Co_0.1_O_2_ (NMC811), was supplied by Ningbo Ronbay Lithium Battery Materials Co. (Ningbo, China). Anode active materials, DXB8 Si/C composite and S360 graphite, were provided by BTR New Material Group Co. (Shenzhen, China). Conductive additives, Super P C45, Super P C65, and GLNA carbon nanotubes, carboxymethyl cellulose (CMC), styrene-butadiene rubber (SBR), and N-methyl-2-pyrrolidone (NMP) were sourced from Gelon Lib Group Co. (Dongguan, China). Polyvinylidene fluoride (PVDF) was obtained from Solvay (Brussels, Belgium). The ceramic-coated separator was purchased from Hebei Gellec New Energy Science&Technology Joint Stock Co. (Handan, China).

### 4.2. Methods

#### 4.2.1. Synthesis of HFEPN

A mixture of sodium metal (617.4 mmol, 14.2 g) and 2,2,2-trifluoroethanol (618 mmol, 46.66 mL) in dry tetrahydrofuran (THF, 240 mL) was stirred under an argon atmosphere at room temperature for 2 h. After the sodium was completely dissolved, the resulting solution was cooled to 10–15 °C. A solution of 2,2,4,4,6,6-hexachlorocyclotriphosphazene (103 mmol, 35.8 g) in THF (240 mL) was added dropwise while maintaining the temperature below 15 °C. The reaction mixture was stirred for 12 h, then quenched with water (25 mL). The THF was removed under reduced pressure, and the resulting residue was diluted with additional water (50 mL) and extracted with dichloromethane (3 × 100 mL). The combined organic extracts were dried over Na_2_SO_4_, filtered, and concentrated to afford a white powder. The crude product was recrystallized from a pentane/diethyl ether (30:1 *v*/*v*) mixture to yield pure HFEPN as a white powder (65.9 g, 87%). The product was dried under vacuum (1 mmHg) at room temperature for 12 h prior to use.

NMR spectra were obtained on a Bruker Avance III HD (Billerica, MA, USA) (400 MHz ^1^H, 101 MHz ^13^C, 376 MHz ^19^F, 162 MHz ^31^P, 40 MHz ^15^N). The chemical shifts are frequency referenced relative to the residual undeuterated solvent peaks. The multiplicity of the signals is indicated as ‘‘s”, ‘‘d”, “t” or ‘‘m” for singlet, doublet, triplet or multiplet, respectively. FT-IR spectra were obtained in a Bruker “Alpha-T” FTIR (KBr) (Billerica, MA, USA).

^1^H NMR (400 MHz, CDCl_3_): δ 4.37−4.18 (m, 12H, –CH_2_–).

^19^F NMR (376 MHz, CDCl_3_): δ −75.39 (s, 18F, –CF_3_).

^31^P NMR (162 MHz, CDCl_3_): δ 16.9 (s, 3P).

^15^N NMR (40 MHz, CDCl_3_): δ 64.7 (m, 3N).

FT-IR (υ/cm^−1^): 1455 (VW), 1421 (VW), 1282 (W), 1236 (M), 1160 (S), 1069 (VS), 961 (S), 886 (W), 847 (W), 795 (M), 655 (W).

#### 4.2.2. Electrolyte Composition

The commercially available reference electrolyte, 1 M LiPF_6_ in an EC/EMC (1:3 *v*/*v*) mixture with 1 wt% VC, was used. A series of electrolytes with the fixed content of the synthesized HFEPN (5, 10, and 15 wt%) were prepared by simple dissolution process, during which the calculated amount of pure LiPF_6_ was added to the mixtures in order to maintain a constant lithium salt concentration and compensate for the dilution introduced by the HFEPN additive.

#### 4.2.3. Physicochemical Characterization of Electrolytes

The ionic conductivity of the prepared electrolytes was evaluated using a Hydromaster Portable meter (HM Digital, Signal Hill, CA, USA). The measurements were performed in a glass vial under a constant argon atmosphere to prevent moisture absorption following the order of increasing HFEPN concentration. The viscosity of the electrolytes was measured using a capillary glass viscometer (VPZh-2m 0.56, Labtex, Moscow, Russia) at 30 °C inside an Ar-filled glovebox (Vilitek, Moscow, Russia).

The electrochemical stability window of electrolytes was determined by cyclic voltammetry (CV). Experiments were performed in an Ar-filled glove box using SP-200 potentio/galvanostat (Biologic, Grenoble, France), in a three-electrode electrochemical cell (electrolyte volume ca. 2.5 mL) with a Pt disk working electrode (diameter 3 mm) and Li foil as both reference and counter electrodes. The potential was swept at a rate of 5 mV/s.

The flame-retardant properties of the electrolytes were evaluated by a self-extinguishing time (SET) test. A silica fiber wick (4 cm in length, 0.8 cm in diameter) was fully saturated with an electrolyte (approx. 0.8 g) and then exposed to a butane lighter flame for 2 s. The burning time was defined as the period from the first appearance of a self-sustaining flame upon removal of the ignition source until its complete extinction using video analysis. The SET value was calculated by normalizing burning time (s) to sample mass (g). Visual documentation of the combustion tests for all concentrations is provided in Supplementary Video S1.

#### 4.2.4. Electrode and Cell Fabrication

The cathode slurry was prepared by mixing 94 wt% LiNi_0.8_Mn_0.1_Co_0.1_O_2_ (NMC811), 3 wt% carbon black (Super P C45), and 3 wt% polyvinylidene fluoride (PVDF) binder in N-methyl-2-pyrrolidone (NMP). The slurry was double-side-coated onto 14 um aluminum foil current collector using doctor blade technique (Zehnter GmbH, Burgdorf, Switzerland), followed by drying at 50 °C for 4 h under active vacuum. In order to prepare Z-fold type battery the dried electrodes were then calendared and laser-cut into 44 × 49 mm sheets with an 8 × 9 mm bare metal tab.

The Si/C composite anode slurry consisted of 94 wt% DXB8 Si/C composite, 1 wt% carboxymethyl cellulose (CMC), 1 wt% carbon black (Super P C65), 1 wt% GLNA carbon nanotubes, and 3 wt% styrene-butadiene rubber (SBR) binder in deionized water. The DXB8 composite is a commercial material. To characterize its morphology, the pristine powder was examined by scanning electron microscopy (SEM) with energy-dispersive X-ray spectroscopy (EDX) (SEM-69-LV, BiOptic; EDX Xplore30, Oxford Instruments, Moscow, Russia). The micrographs (see Figure 4) reveal a morphology typical for such composites, with uniform dispersion of fine silicon particles in a carbon matrix. The graphite anode slurry was prepared similarly, using 94% S360 graphite, 1 wt% CMC, 2 wt% carbon black, and 3 wt% SBR. Both slurries were double-side-coated onto 8 μm copper foil, dried at 70 °C, calendared, and laser-cut into 46 × 51 mm electrodes with an 8 × 7 mm bare nickel tab. All electrodes were finally dried under vacuum at 70 °C for 12 h prior to cell assembly. The mass loading of the cathode and anode active materials was calculated to achieve a total capacity of 1 Ah for a cell stack comprising five cathodes and six anode sheets.

Pouch Cell Assembly: Multi-layer electrode stacks, consisting of five cathodes and six anodes separated by a ceramic-coated separator, were assembled using a stacking machine (TOB, Xiamen, China). Aluminum and nickel tabs were welded to the cathode and anode stacks, respectively, using an ultrasonic welder (Gelon, Dongguan, China). The stacks were then heat-sealed into aluminum-laminate pouch bags using a sealer (Kejing Star, Shenzhen, China). These pre-assembled pouches were dried at 70 °C under vacuum for 12 h.

The final assembly was conducted in an argon-filled glovebox (H_2_O, O_2_ < 0.1 ppm). Each pouch cell was filled with 4 mL of electrolyte and sealed. To ensure complete electrolyte impregnation, the sealed cells were clamped between flat plates. For Si/C-based cells, an additional degassing procedure was implemented: cells were carefully re-opened in the glovebox antechamber under active vacuum for 20 min to remove gases from material pores, then resealed and rested in a vacuum oven at 50 °C for 4 h.

#### 4.2.5. Electrochemical Testing

Galvanostatic cycling tests were performed using a Neware BTS-4008 battery test system (Shenzhen, China). All formation and cycling tests were conducted within a voltage window of 2.7–4.2 V at room temperature using CCCV mode upon charge and CC mode upon discharge. The formation protocol varied by anode chemistry: Si/C composite cells were cycled at 0.05C current for 2 cycles, while graphite-based cells were cycled at 0.05C for 10 h, followed by 0.1C for the remainder of the first cycle and 5 subsequent cycles. Electrochemical impedance spectroscopy (EIS) was employed to investigate solid–electrolyte interphase (SEI) formation in specially assembled Li||Si/C coin cells after one discharge–charge cycle at 0.1C. These cells were assembled with the Si/C composite as the working electrode and lithium metal as the counter/reference electrode, impregnated with the target electrolyte mixtures, and kept at room temperature overnight. After one full discharge–charge cycle at a 0.1C rate, EIS measurements were conducted. The spectra were recorded using a Corrtest CS310X (Wuhan, China) potentiostat-galvanostat in the frequency range from 100 kHz to 0.01 Hz with an amplitude of 5 mV (7 points per decade). Prior to each EIS measurement, the cell was held at the target voltage for 30 min (chronoamperometry) to establish a steady-state potential. Specific surface area of the DXB8 Si/C material is 3 m^2^/g according the vendor.

## 5. Conclusions

This study demonstrates the dual functionality of hexakis(fluoroethoxy)phosphazene (HFEPN) as a flame-retardant electrolyte additive for lithium-ion batteries. A key finding is that its flame-retardant efficacy exhibits a distinct threshold behavior, with non-flammability achieved at a 15 wt% concentration in our electrolyte system, a value influenced by solvent composition and test conditions. Concerning electrochemical performance, HFEPN facilitates the formation of a more conductive solid–electrolyte interphase (SEI) on silicon-graphite composite anodes, as evidenced by a significant enhancement in interfacial kinetics. However, this interfacial benefit is counterbalanced by a concentration-dependent increase in electrolyte viscosity, which imposes mass-transport limitations on cell performance, particularly under high-rate cycling.

Consequently, the use of HFEPN represents a trade-off between safety and performance. While it provides substantial flame-retardant capabilities without introducing parasitic reactions, its optimal application may be in systems where operational currents are moderate, and safety enhancements are prioritized. This work highlights that while HFEPN itself shows limited stabilizing effect under strenuous conditions, the phosphazene core remains a highly promising scaffold for designing advanced electrolyte components. Future efforts should focus on molecular engineering—for instance, developing liquid, polymerizable phosphazene derivatives—to decouple the beneficial flame-retardant and SEI-forming functions from the detrimental effects on electrolyte transport properties. This work highlights the importance of considering both interfacial and bulk transport properties when designing multifunctional electrolyte systems for next-generation batteries.

## Data Availability

The original contributions presented in this study are included in the article and Supplementary Materials. Further inquiries can be directed to the corresponding author.

## Supplementary Materials

The following supporting information can be downloaded at https://www.mdpi.com/article/10.3390/ijms27010028/s1.

## Appendix A

**Table A1:** Physicochemical data (viscosity, ionic conductivity) of the reference electrolyte and HFEPN-containing mixtures.

Mixture	Viscosity (cP)	Ionic Conductivity (mS/cm)
Reference electrolyte	2.77	8.40
5 wt% HFEPN	3.15	7.60
10 wt% HFEPN	3.62	6.82
15 wt% HFEPN	3.90	6.11
